# Supplementary material for: Noncoding genetic variation in ISPD distinguishes gamecocks from nongame chickens
Source: bioRxiv. 2023 Aug 21:2023.08.16.553562. Preprint. [Version 1] doi: 10.1101/2023.08.16.553562 (PMC10473654; doi:10.1101/2023.08.16.553562)
Supplement: 1 [file NIHPP2023.08.16.553562V1-supplement-1.pdf]

bioRxiv preprint doi: https://doi.org/10.1101/2023.08.16.553562; this version posted August 21, 2023. The copyright holder for this preprint (which was not certified by peer review) is the author/funder, who has granted bioRxiv a license to display the preprint in perpetuity. It is made available under aCC-BY 4.0 International license.

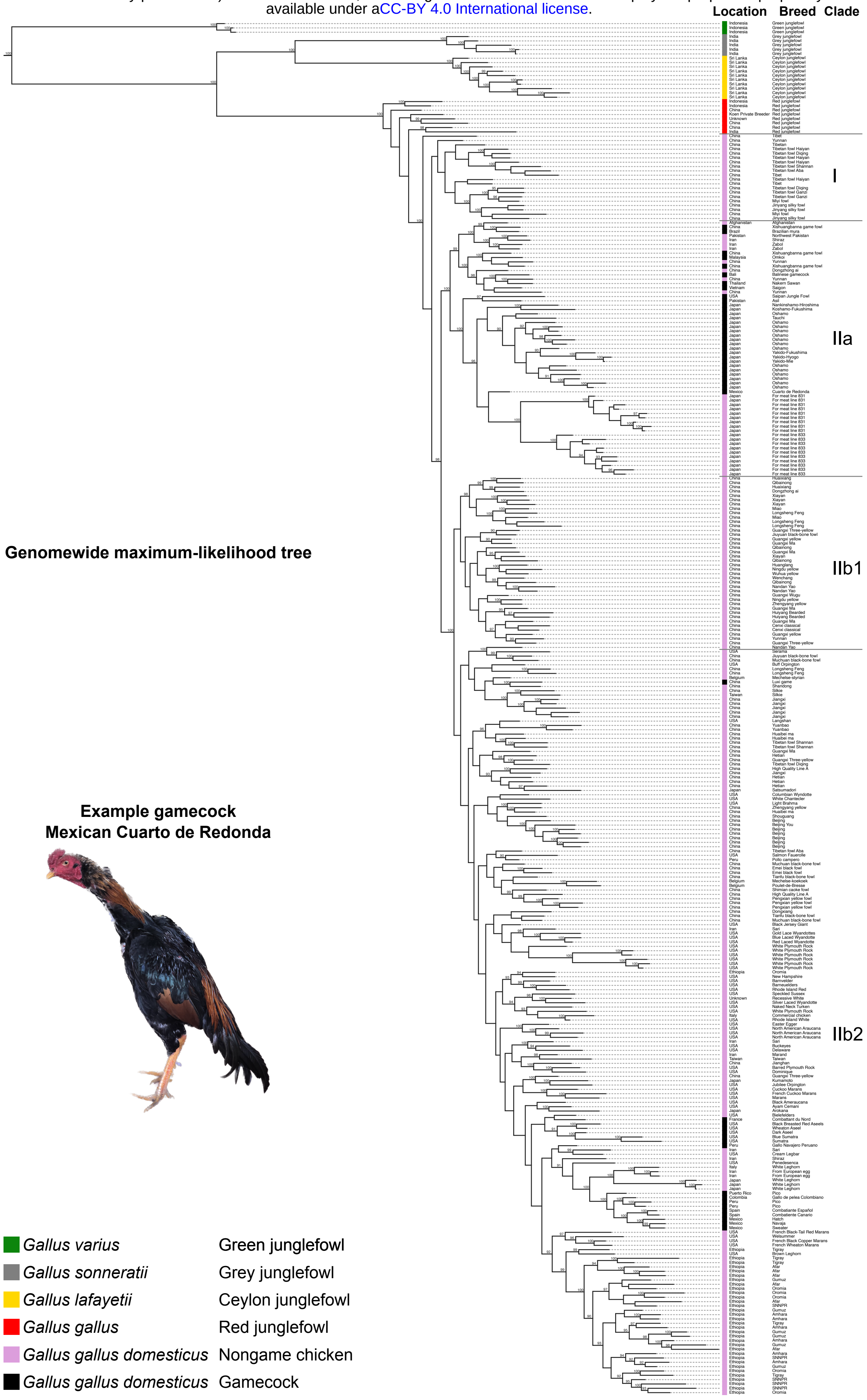

bioRxiv preprint doi: https://doi.org/10.1101/2023.08.16.553562; this version posted August 21, 2023. The copyright holder for this preprint (which was not certified by peer review) is the author/funder, who has granted bioRxiv a license to display the preprint in perpetuity. It is made available under aCC-BY 4.0 International license.

Suppl. Figure 1. Genome-wide phylogenetic tree of *Gallus* including chickens

Maximum-likelihood phylogenetic tree based on whole-genome data, including all species in the junglefowl (*Gallus*) genus, as well as gamecocks and nongame chickens from around the world. The name and geographic origin of the samples is presented on the right. Bootstrap support values ≥90 are highlighted on the branches.

bioRxiv preprint doi: https://doi.org/10.1101/2023.08.16.553562; this version posted August 21, 2023. The copyright holder for this preprint (which was not certified by peer review) is the author/funder, who has granted bioRxiv a license to display the preprint in perpetuity. It is made available under aCC-BY 4.0 International license.

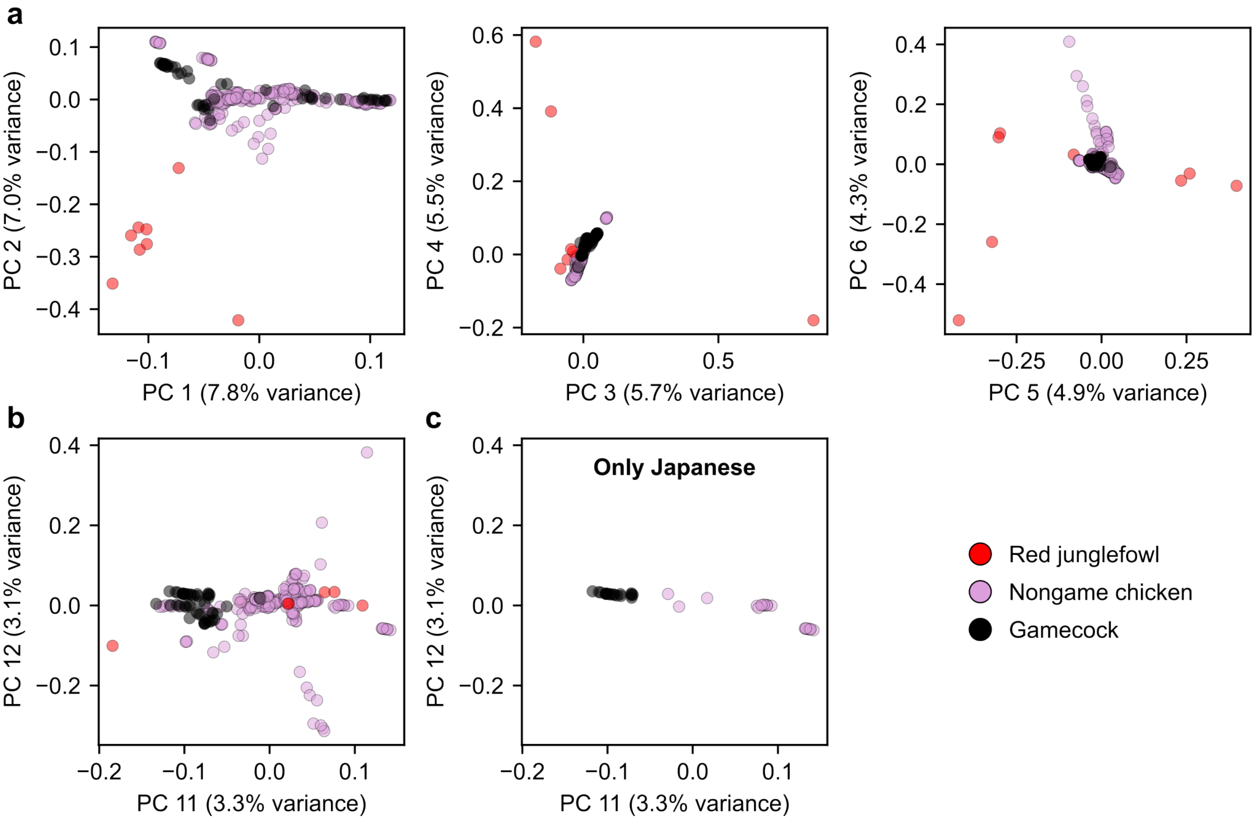

Suppl. Figure 2. PCA of genetic variation in *Gallus gallus* samples.

a, PC 1 and 2 separate wild Red junglefowl from domesticated chickens (nongame chickens and gamecocks). **b,** PC 11 mostly separates chickens into nongame chickens and gamecocks. **c,** Same as **b** but including Japanese samples exclusively.

bioRxiv preprint doi: https://doi.org/10.1101/2023.08.16.553562; this version posted August 21, 2023. The copyright holder for this preprint (which was not certified by peer review) is the author/funder, who has granted bioRxiv a license to display the preprint in perpetuity. It is made available under aCC-BY 4.0 International license.

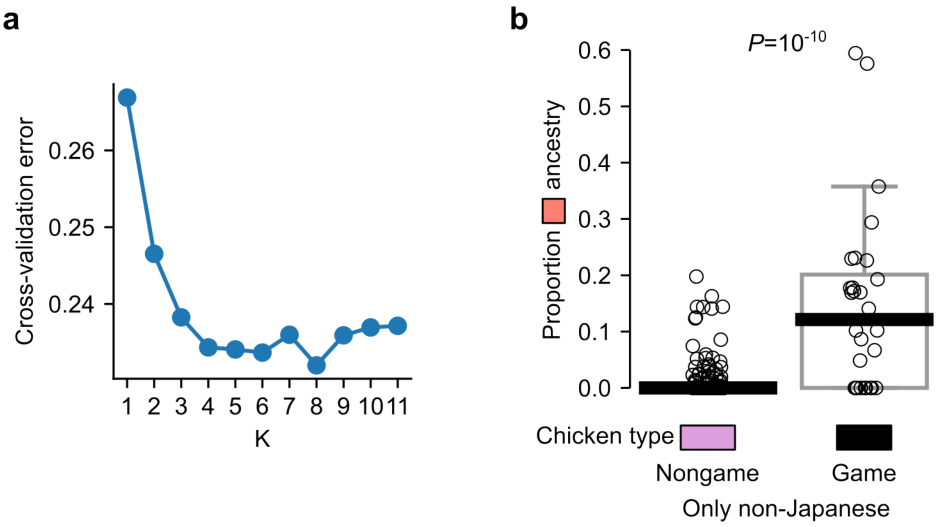

Suppl. Figure 3. ANCESTRY cross-validation error and analysis without Japanese samples

a, Cross-validation error at different values of K. **b,** Proportion "salmon-colored" ancestry in samples that are not from Japan.

bioRxiv preprint doi: https://doi.org/10.1101/2023.08.16.553562; this version posted August 21, 2023. The copyright holder for this preprint (which was not certified by peer review) is the author/funder, who has granted bioRxiv a license to display the preprint in perpetuity. It is made available under aCC-BY 4.0 International license.

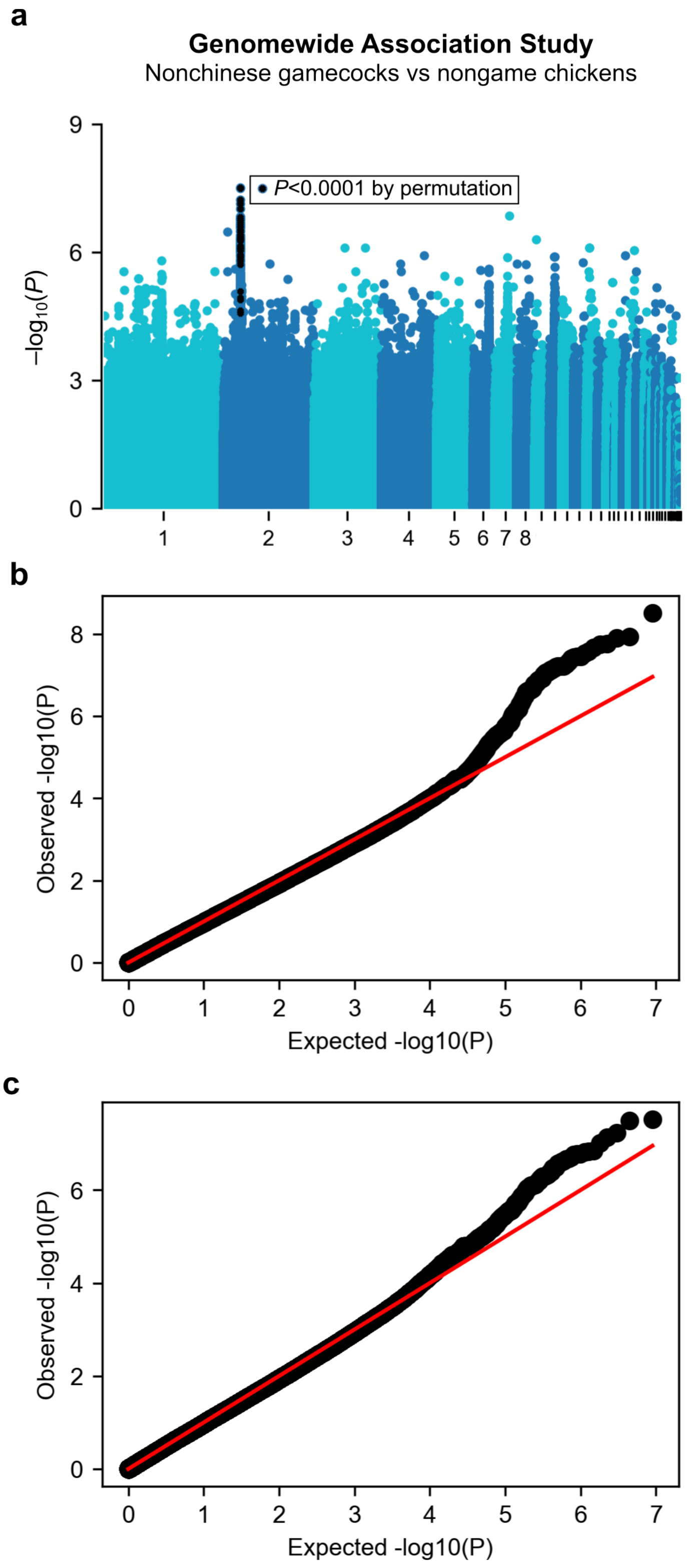

Suppl. Figure 4. Genome-wide association study excluding Chinese chickens and Q-Q plots

a, GWAS of gamecocks that are not from China (n=44) vs nongame chickens (n=62). *P*-values on the y axis using genomic control. Black denotes variants with $P<10^{-4}$ by permutation; no variants outside chromosome 2 surpassed that permutation threshold. **b,** Q-Q plot of GWAS in Figure 2a. **c,** Q-Q plot of GWAS in panel **a** of this figure.

bioRxiv preprint doi: https://doi.org/10.1101/2023.08.16.553562; this version posted August 21, 2023. The copyright holder for this preprint (which was not certified by peer review) is the author/funder, who has granted bioRxiv a license to display the preprint in perpetuity. It is made available under aCC-BY 4.0 International license.

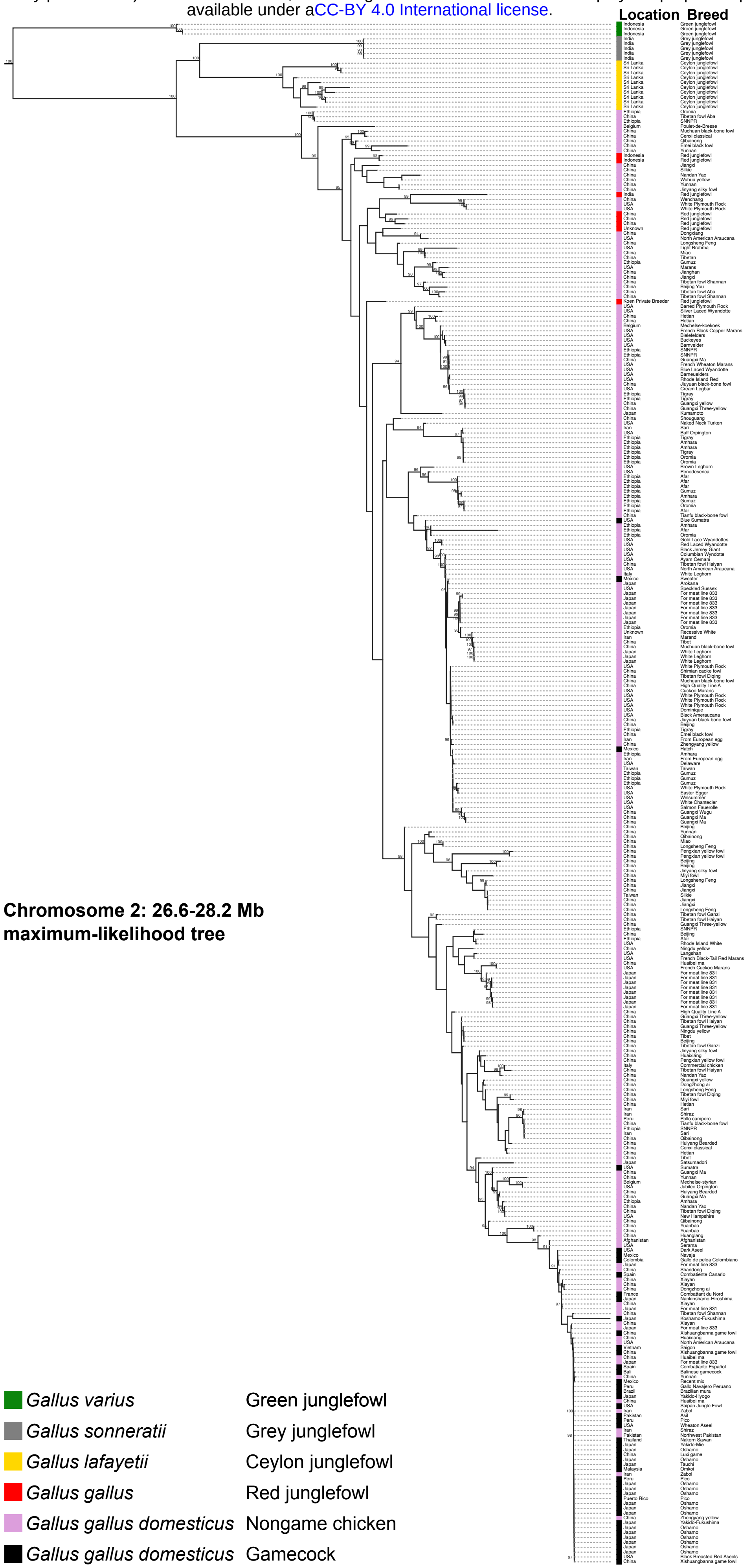

bioRxiv preprint doi: https://doi.org/10.1101/2023.08.16.553562; this version posted August 21, 2023. The copyright holder for this preprint (which was not certified by peer review) is the author/funder, who has granted bioRxiv a license to display the preprint in perpetuity. It is made available under aCC-BY 4.0 International license.

Suppl. Figure 5. Phylogenetic tree of Chromosome 2 PBS locus that distinguishes gamecocks from nongame chickens

Maximum-likelihood phylogenetic tree of Chromosome 2 locus (26.6–28.2 Mb), including all species in the junglefowl (*Gallus*) genus, as well as gamecocks and nongame chickens from around the world. The name and geographic origin of the samples is presented on the right. Bootstrap support values ≥90 are highlighted on the branches.

bioRxiv preprint doi: https://doi.org/10.1101/2023.08.16.553562; this version posted August 21, 2023. The copyright holder for this preprint (which was not certified by peer review) is the author/funder, who has granted bioRxiv a license to display the preprint in perpetuity. It is made available under aCC-BY 4.0 International license.

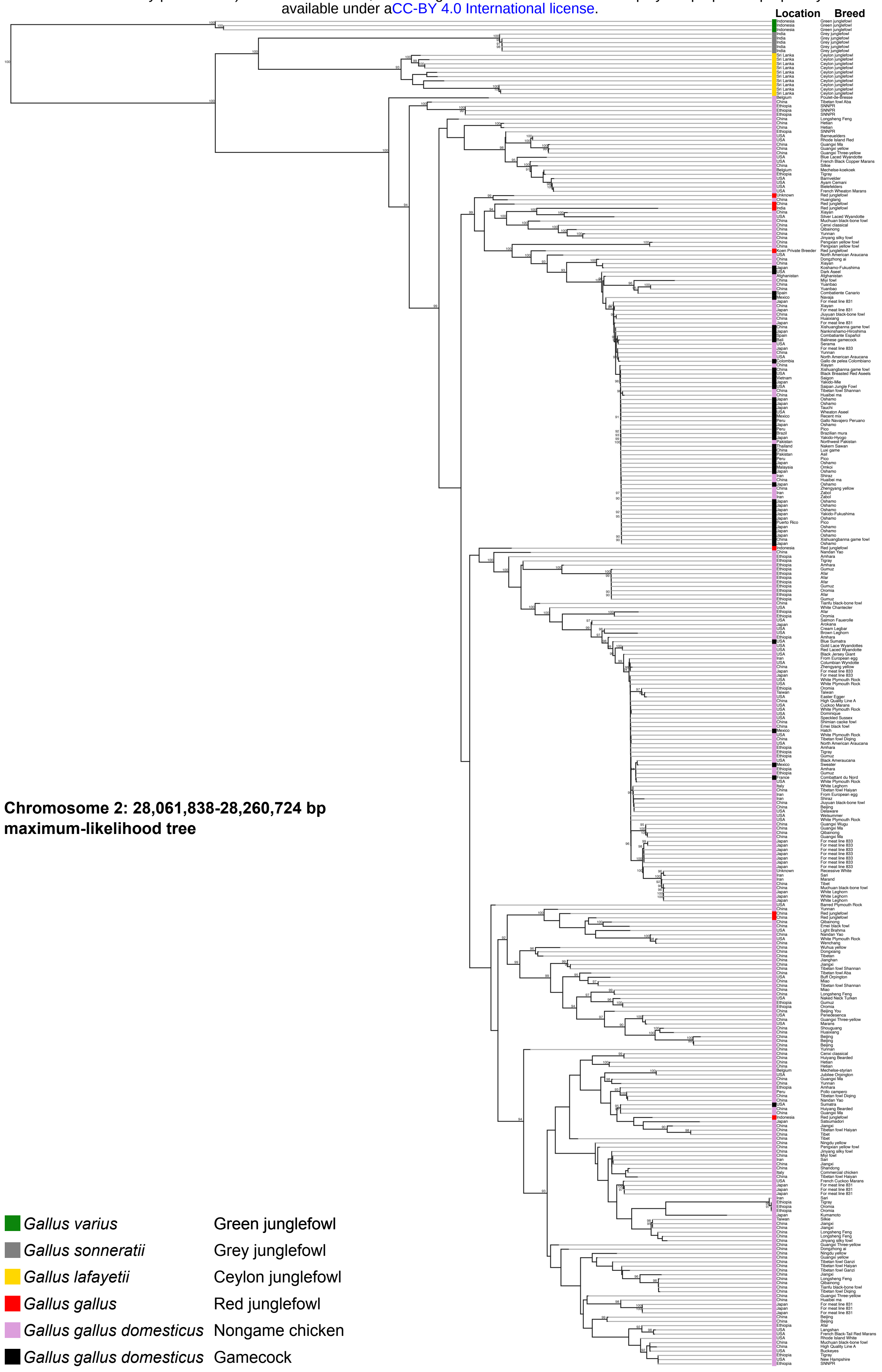

bioRxiv preprint doi: https://doi.org/10.1101/2023.08.16.553562; this version posted August 21, 2023. The copyright holder for this preprint (which was not certified by peer review) is the author/funder, who has granted bioRxiv a license to display the preprint in perpetuity. It is made available under aCC-BY 4.0 International license.

Suppl. Figure 6. Phylogenetic tree of Chromosome 2 GWAS locus that distinguishes gamecocks from nongame chickens

Maximum-likelihood phylogenetic tree of Chromosome 2 locus (28,061,838–28,260,724 bp), including all species in the junglefowl (*Gallus*) genus, as well as gamecocks and nongame chickens from around the world. The name and geographic origin of the samples is presented on the right. Bootstrap support values ≥90 are highlighted on the branches.

bioRxiv preprint doi: https://doi.org/10.1101/2023.08.16.553562; this version posted August 21, 2023. The copyright holder for this preprint (which was not certified by peer review) is the author/funder, who has granted bioRxiv a license to display the preprint in perpetuity. It is made available under aCC-BY 4.0 International license.

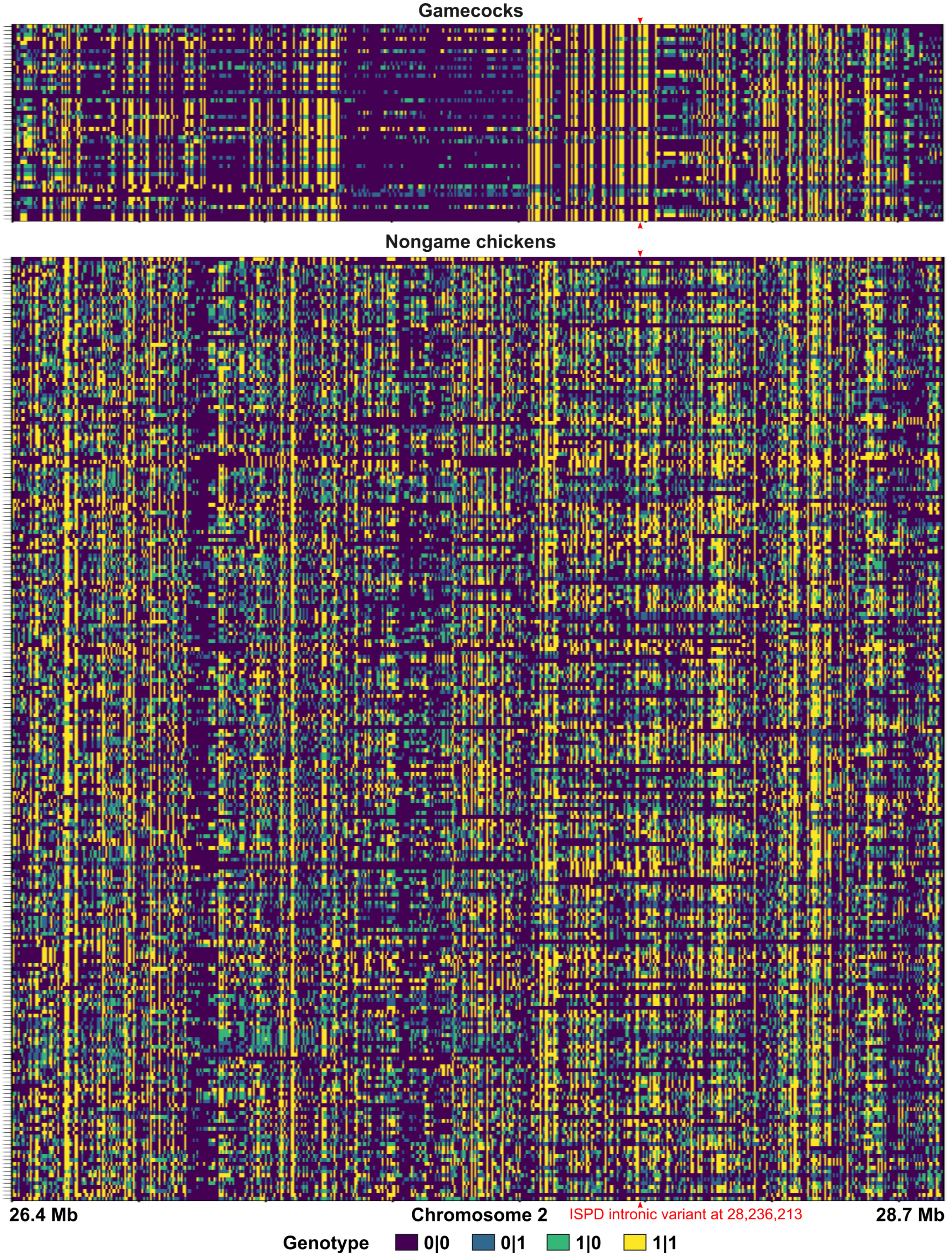

Suppl. Figure 7. Haplotypes of Chromosome 2: 26.4–28.7 Mb in gamecocks and nongame chickens
